# Altered MicroRNA Expression in Bovine Subcutaneous and Visceral Adipose Tissues from Cattle under Different Diet

**DOI:** 10.1371/journal.pone.0040605

**Published:** 2012-07-10

**Authors:** Josue Moura Romao, Weiwu Jin, Maolong He, Tim McAllister, Le Luo Guan

**Affiliations:** 1 Department of Agricultural, Food and Nutritional Science, University of Alberta, Edmonton, Alberta, Canada; 2 Lethbridge Research Centre, Agriculture and Agri-Food Canada, Lethbridge, Alberta, Canada; Nottingham University, United Kingdom

## Abstract

**Background:**

MicroRNAs (miRNAs) are a class of molecular regulators found to participate in numerous biological processes, including adipogenesis in mammals. This study aimed to evaluate the differences of miRNA expression between bovine subcutaneous (backfat) and visceral fat depots (perirenal fat) and the dietary effect on miRNA expression in these fat tissues.

**Methodology/Principal Findings:**

Fat tissues were collected from 16 Hereford×Aberdeen Angus cross bred steers (15.5 month old) fed a high-fat diet (5.85% fat, n = 8) or control diet (1.95% fat, n = 8). Total RNA from each animal was subjected to miRNA microarray analysis using a customized Agilent miRNA microarray containing 672 bovine miRNA probes. Expression of miRNAs was not equal between fat depots as well as diets: 207 miRNAs were detected in both fat depots, while 37 of these were found to be tissue specific; and 169 miRNAs were commonly expressed under two diets while 75 were diet specific. The number of miRNAs detected per animal fed the high fat diet was higher than those fed control diet (p = 0.037 in subcutaneous fat and p = 0.002 visceral fat). Further qRT-PCR analysis confirmed that the expression of some miRNAs was highly influenced by diet (miR-19a, -92a, -92b, -101, -103, -106, -142–5p, and 296) or fat depot (miR-196a and -2454).

**Conclusions/Significance:**

Our results revealed that the miRNA may differ among adipose depots and level of fat in the diet, suggesting that miRNAs may play a role in the regulation of bovine adipogenesis.

## Introduction

Adipose tissue is dynamic, with its fundamental activity in the regulation of energy balance, and its role in endocrine function becoming increasingly evident [1], [2]. Adipogenesis is the process by which preadipocytes differentiate into adipocytes [3]. The extent of adipogenesis is influenced by a number of factors including diet, fat depot, age and breed [4], [5], [6]. Studies have shown the importance of adipogenic transcription factors (PPARγ, C/EBPs, KLFs and SERBP), which regulate the expression of many adipogenic genes that participate in adipocyte differentiation [7], [8].

A class of gene regulators known as microRNAs (miRNAs) have been discovered to regulate gene expression in many biological processes including embryo development, differentiation, apoptosis, and metabolism in animals [9], [10], [11]. These molecules are small non-coding RNAs with approximately 22 nucleotides that are able to repress gene expression, by binding to messenger RNAs in a sequence-specific manner [12]. Such regulatory roles appear to be tissue specific as many tissue specific miRNAs have been identified [13], [14]. Recent studies reported that adipogenesis in humans and mice is also regulated by miRNAs, with several miRNAs being reported to have pro or anti-adipogenic roles [15], [16] through repression of various genes, including transcriptional factors such as PPARγ, PPARα and KLF5 [17], [18], [19], [20]. MiRNAs have also been shown to be differentially expressed in bovine adipose tissue with the expression of mir-378 expression varying with thickness of subcutaneous fat [21]. This miRNA is also differentially expressed in murine adipocytes during differentiation [22] and its pro-adipogenic activity is possibly through regulation of two tumour suppressor genes, SUFU and FUS-1 genes [23].

Recent studies have revealed that changes in the energy density of the diet influences gene expression in adipose tissue [24], [25]. However, it is not known if altering level of dietary energy by changing lipid levels influences miRNA expression in bovine adipose tissue. We hypothesized that miRNA expression differs between adipose depots and that level of lipids in the diet alters miRNAs expression in these depots. Therefore, this research aimed to determine the miRNA expression profile from subcutaneous and visceral adipose depots of beef cattle fed diets containing high or low levels of dietary fat.

## Results

### Performance and Fat Traits of Steers

Feeding steers with diets containing varying lipid content affected phenotypic traits of steers. Animals fed High fat diet had a higher body weight gain as well as an improved feed efficiency (Table 1). Despite statistical comparisons for fat traits (cutability, backfat thickness and adipocyte size) between control and high fat group there was only a trend in for steers fed the high fat diet to have lower cutability, higher backfat thickness and greater adipocyte size (Table 1).

**Table 1:** Performance and fat traits of animals fed control or high fat diet.

	Control	High fat	
Performance andfat traits	Mean ± SD	Mean ± SD	p-value
Weight gain (kg)	151.70±20.69	181.40±24.06	0.018
Feed Conversion Rate	5.76±0.51	5.20±0.30	0.019
Cutability (%)	53.25±2.66	51.13±1.64	0.075
Backfat thickness (mm)	17.62±5.01	20.75±3.54	0.171
Adipocyte size (µm)*	134.71±8.95	146.50±13.78	0.062

* Adipocytes used for analysis were derived from backfat tissue.

### Microarray Analysis of miRNAs Expression under Different Diets

From a total of 672 miRNA probes tested in the miRNA microarray, 244 were expressed in adipose tissue from at least one animal (Table S1). The total number of miRNAs expressed in each group of steers was 207 for control and 206 for High fat group. When the profiles of miRNA were compared, a total of 169 miRNAs were simultaneously expressed in steers fed the high fat and control diet. A total of 75 miRNA were diet specific in subcutaneous or visceral fat, as 43 were detected only in the Control diet and 31 exclusively in the High fat diet, from subcutaneous adipose tissue. While for visceral adipose tissue, 31 miRNAs were detected only steers fed the Control and 37 miRNAs in those fed the High fat.

The number of miRNAs detected from each steer, ranged from 115 to 162, for Control group, and 162–163 for High fat group. More miRNAs were detected in cattle fed the high fat diet (p = 0.037 in subcutaneous fat and p = 0.002 in visceral fat) than in those fed the control diet. Steers fed the high fat diet had a lower variability in the number of miRNA expressed than control steers (Table 2).

**Table 2:** miRNAs detected by individual and average according to diet and fat tissue.

	Control diet			High fat diet	
ID	Subcutaneous fat	Visceralfat	ID	Subcutaneous fat	Visceralfat
C108	159	126	F103	163	162
C111	131	115	F109	162	163
C209	162	124	F202	162	163
C211	160	153	F204	162	162
C307	160	129	F302	163	163
C308	142	128	F303	163	162
C405	118	161	F411	162	163
C410	159	162	F412	163	163
Avg.	148.8±16.6^b^	137.2±18.4^b^		162.5±0.5^a^	162.6±0.5^a^

a,b different letters mean significant difference between diet or fat depot comparisons, p<0.05.

### Microarray Analysis of miRNAs Expression under Different Fat Depots

A total of 207 miRNAs were detected in both fat depots, while 37 of these were found to be tissue specific. Control steers had 15 miRNAs detected solely in subcutaneous fat and 16 solely in visceral fat. Steers fed the high fat diet had 8 miRNAs solely detected in subcutaneous fat and 27 miRNAs in visceral fat. There was no statistical difference in the average number of miRNAs detected in different fat depots in steers fed either diet (Table 2).

### Frequency of miRNAs Detected in the Experimental Population

**Figure 1:**
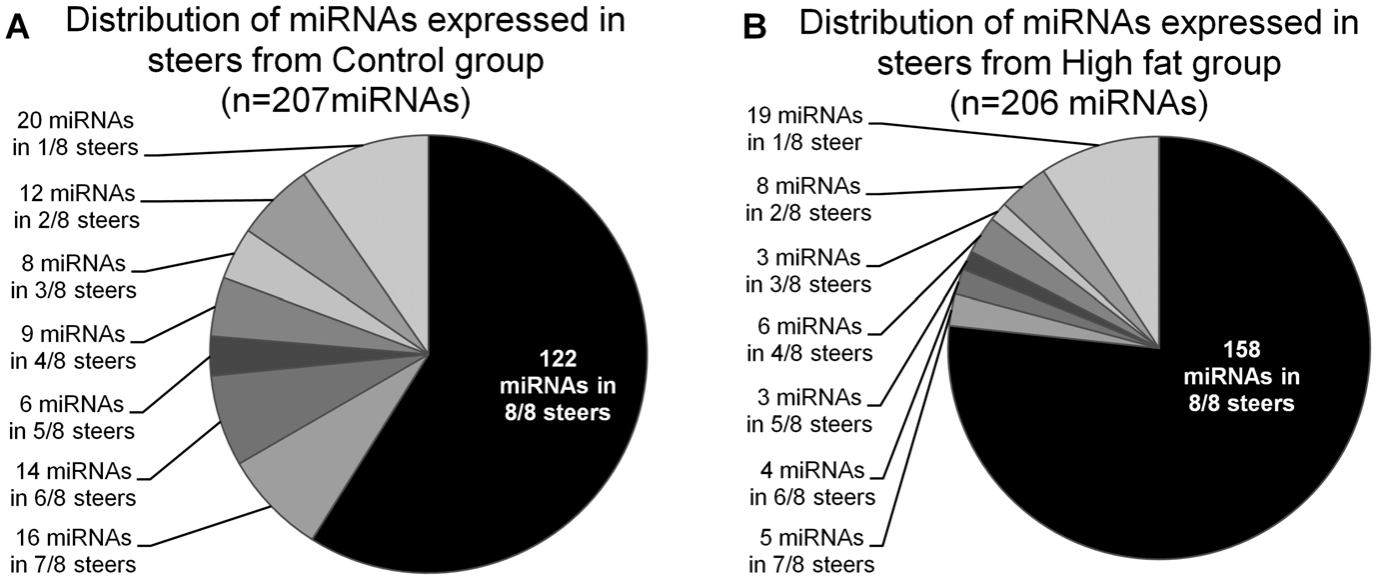
Distribution of miRNAs detected in steers fed control diet (A) or high fat diet (B). The distribution of miRNAs considers data from both subcutaneous and visceral fat tissue for Control (A) and High fat diet (B) groups. Each pie chart represents how the total amount of miRNAs (Control = 207, High fat = 206) are distributed within the animal population of each group, varying from a maximum of eight out of eight animals to a minimum of one out of eight.

The miRNAs detected varied from being present in only a single steer on each diet to being present in all eight animals sampled. For example, we detected only 118 miRNAs in subcutaneous fat tissue in a steer (ID: C405) fed the control diet while in another steer (ID: C209) 162 miRNAs were detected. The majority of miRNAs (58.9% in Control and 76.7% in High fat) detected were expressed in all eight animals from each treatment, while the remainder of miRNAs (41.1% in Control and 23.3% in High fat) were detected in from one up to seven animals (Figure 1). MiRNAs detected from steers fed the high fat diet, regardless of the depot, were more consistently detected than in steers from the Control group. A total 122 of miRNAs detected in control group were conserved in all eight steers, while 158 miRNAs from High fat group were conserved in all steers. Despite differences in miRNA profiles due to diet or tissue-specificity, a total of 83 miRNAs were simultaneously expressed in both fat depots and steers fed either the high fat or control diet.

### MiRNAs Selected for qRT-PCR Validation

Twelve miRNAs were selected for qRT-PCR validation based on miRNA microarray expression data (Table S1). They presented significant differences (p<0.05) among the 4 experimental categories, consisting of combinations of two diets with two fat depots (miR-16b, -19a, -92a, -92b, -101, -103, -106, -142–5p, -196a, -296, -2368* and, -2454).

**Figure 2:**
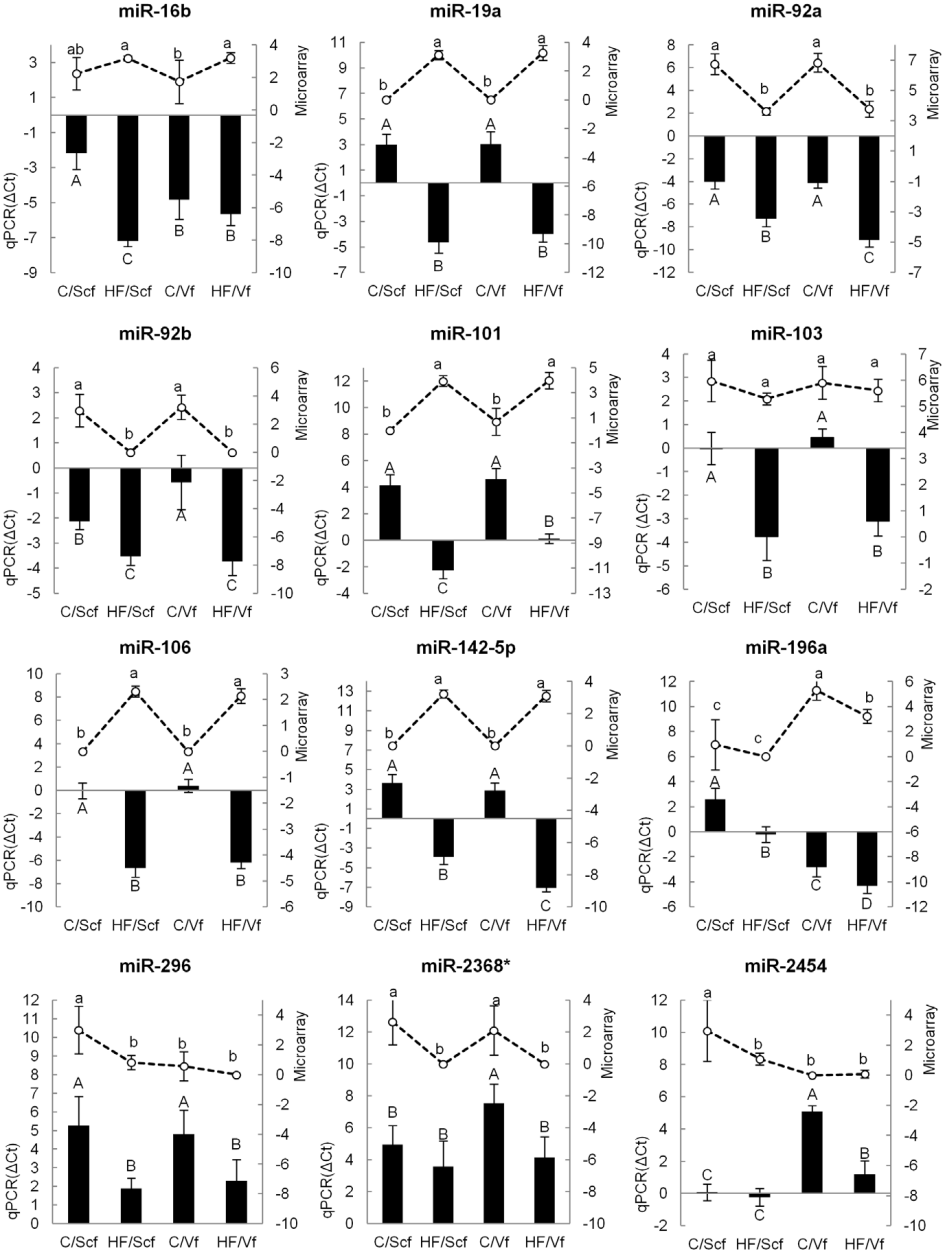
Expression of miRNAs by qRT-PCR and microarray. MicroRNA expression is presented for the following treatments on the horizontal axis: Control diet/Subcutaneous fat (C/Scf), High fat diet/Subcutaneous fat (HF/Scf), Control diet/Visceral fat (C/Vf) and High fat diet/Visceral fat (HF/Vf). The graphs show the miRNA expression from miRNA microarray represented by lines (–○–) on the top and values are shown on the left vertical axis as normalized intensity values. Quantitative PCR expression is represented using columns (←) on the bottom and values are shown on the right vertical axis as delta cycle threshold (ΔCt). A, B, C, D Columns (qPCR) with different letters differ significantly (p<0.05). a, b, c Markers in lines (microarray) with different letters differ significantly (p<0.05). Data are presented as Mean ± Standard deviation.

The expression of 8 miRNAs (miR-19a, -92a, -92b, -101, -103, -106, -142–5p, and 296) was notably higher in steers fed the high fat diet than those fed the control (Figure 2). Increases ranged from a low of 2.62 fold in subcutaneous adipose tissue and 8.94 fold in the visceral adipose tissue for miR-92b, to a high of 185.11 in subcutaneous adipose tissue and 968.77 fold in the visceral adipose tissue for miR-142–5p. Expression of other miRNAs was heavily influenced by fat depot. MiR-2454 expression was higher in subcutaneous fat compared to visceral fat (32.20 fold in Control and 2.67 fold in high fat) whereas, conversely, miR-196a had a higher expression in visceral than subcutaneous fat (43.06 fold in Control and 17.25 fold in High fat). The miR-2368* and miR-16b also exhibited significant differences in expression, however their changes between diet types were not detected in either fat depot, with miR-16b being observed only in subcutaneous fat and miR-2368* in visceral fat (Figure 2). The comparison between qRT-PCR and microarray expressions showed that they were not showing the same trends for all miRNAs. Six miRNAs (miR-16b, miR-19a, miR-106, miR142–5 p, miR-196a and miR-2454) had the expression in agreement between qRT-PCR and microarray, however the other six (miR92a, miR-92b, miR-101, miR-103, miR-296 and miR-2368*) had divergent results.

### MiRNA Predicted Targets and Functional Analysis

A total of 34 unique genes expressed in adipose tissue having functions related to lipid metabolism or adipogenesis were predicted to be targets of the 12 miRNAs validated by qRT-PCR (Table 3). The amount of potential gene targets varied among miRNAs with a high of seven predicted genes for miR-101 and miR-2368* and a low of one predicted gene for miRNA-196a. The majority of the predicted genes (28 out of 34) were predicted targets of only one of the 12 miRNAs analyzed, while six of them (ABHD5, ADRB1, CLOK, PPARGC1B, REST and SGK1) were predicted targets for two miRNAs. The functional analysis identified the biological functions relevant to the 34 predicted target genes (Table 4). A total of 25 biological functions were relevant to adipose tissue physiology, with 24 were classified in the Lipid Metabolism category and 1 in the Connective Tissue Development and Function category. Some biological functions were more prevalent than other, such as synthesis of lipids which accounted for 19 of the 34 of the predicted genes.

## Discussion

Diet is known to impact many phenotypic traits in ruminants such as growth rate, meat and the fat content and fatty composition of milk and meat. Steers fed the high fat diet (5.85%) in the present study had higher weight gain and improved feed efficiency compared to those fed the control diet, with some of these differences likely being attributable to differences in adipose metabolism as suggested by changes in carcass cutability, backfat thickness and adipocyte size. Recent advanced functional genomics approaches have allowed the identification of the mechanisms of adipogenesis at gene expression level [5] and their alteration in response to different diets [24], [26]. A recent study showed that beef steers supplemented with a high fat supplement (0.31 Kg/day of corn oil) presented a higher lipogenic activity in fat tissue, with higher expression of Fatty acid synthase and Stearoyl-CoA desaturase [24]. The increase of adipose mass by high fat diets can also enhance the production of adipokines, influencing the regulation not only of fat tissue, but also promoting systemic effects on metabolism [2], [27]. In this study, we further investigated the molecular regulation of beef cattle adipose tissue by characterizing the expression of miRNAs in adipose tissue.

MiRNAs are known to regulate gene expression by binding to complementary sequences of their target messenger RNAs which leads to translational repression and affects several physiological processes, including adipogenesis [16], [28]. MiRNAs from adipose tissue exhibited differential expression levels between steers of distinct breed as well as with different levels of backfat thickness [21]. A recent study further showed variation of miRNA profiles among different locations of subcutaneous backfat [29]. Our current study is the first to report that the miRNA expression profiles in steers differed significantly in two fat depots in response to varying dietary fat content. Our results are in agreement with another study that showed the manipulation of dietary fat (varying levels of conjugated linoleic acid in the diet) impacted the expression of miRNAs (miR-143, -107, -221 and -222) from retroperitoneal adipose tissue of mice [30]. Although the miRNA expression was altered due to dietary manipulation in both species; mice and bovine have different molecular mechanisms of adipogenesis [31], [32], in part due to variation in the fatty acid profile presented to adipocytes as a result of differences in microbial biohydrogenation in the intestinal tract.

Microarray analysis revealed that most miRNAs were simultaneously expressed in steers fed both diets, but a parcel of miRNAs was specific to each diet which might tailor the regulation of adipose tissue to the specific metabolic requirements imposed by each diet. Dietary fat changes alter gene expression (mRNA level) in bovine adipose tissue [26] and considering that miRNA is a major regulator on gene expression, it is perhaps not unexpected that distinct miRNA profiles in adipose tissue are associated with changes in the fat content of the diet. Steers fed the high fat diet had a higher average number of miRNA expressed compared to control steers (Table 2), suggesting that a high fat diet increased the complexity of the role of miRNAs in regulating gene expression. We speculate that it is likely that all pathways leading to fat development are activated in the adipose tissue of high fat diet animals, therefore more miRNAs are needed to regulate fat development in animals fed high fat diet. However, the differences in miRNA number between steers from control and high fat groups may also suggest that animals in either group have different response to the environment effect or interactions between genetic regulation and environment influence. Future studies to investigate the diet effects on the same animals are necessary to clarify if the expression of microRNAs is directly regulated by the diet.

Two parameters can differentiate the miRNA profile between individuals. One is the presence or absence of distinct miRNAs and the other is the level of expression of each miRNA. However it is expected that individuals should express a core number of miRNAs fundamental for the regulation of genes involved in adipose metabolism. Steers fed high fat diet presented a larger core (76.7%) of miRNAs (expressed in all eight animals) compared to control steers (58.9%), suggesting that a high fat diet demands a larger regulational miRNA core to regulate adipose tissue metabolism. Among those, we detected well studied miRNAs including miR-103 which has a pro-adipogenic role [33], miRNA let-7, reported to have an anti-adipogenic role in 3T3-L1 adipocyte cell culture regulating HMGA2 [34] and miR-27b which regulates the expression of PPARγ, considered the master regulator of adipogenesis [35]. These findings suggest that miRNA based regulation in adipogenesis is common in different mammalian species. It is noticeable that the first bovine specific miRNAs identified may be associated with bovine adipogenesis. For example, miR-2368* and miR-2454 are two bovine specific miRNAs [36]. Although their functions are unknown, based on bioinformatics prediction tools they may regulate mRNAs belonging to peroxisome proliferator-activated receptor alpha (PPARA) and platelet-derived growth factor beta polypeptide (PDGFB) genes. Future studies are necessary to understand their roles in visceral fat in response to different diets.

**Table 3:** Predicted miRNA gene targets with functions related to lipid metabolism and/or adipogenesis.

microRNA	Symbol	Entrez Gene Name
bta-miR-16b	FGF2	fibroblast growth factor 2 (basic)
	GNAI3	guanine nucleotide binding protein (G protein), alpha inhibiting activity polypeptide 3
	LRP6	low density lipoprotein receptor-related protein 6
	PAFAH1B2	platelet-activating factor acetylhydrolase 1b,catalytic subunit 2 (30 kDa)
	SMAD7	SMAD family member 7
	WNT3A	wingless-type MMTV integration site family, member 3A
bta-miR-19a	SOCS3	suppressor of cytokine signaling 3
	SGK1	serum/glucocorticoid regulated kinase 1
	ADRB1	adrenergic, beta-1-, receptor
	ABHD5	abhydrolase domain containing 5
bta-miR-92a,b	ADRB1	adrenergic, beta-1-, receptor
	TEF	thyrotrophic embryonic factor
bta-miR-101	SLC12A2	solute carrier family 12 (sodium/potassium/chloride transporters), member 2
	SGK1	serum/glucocorticoid regulated kinase 1
	PRKCE	protein kinase C, epsilon
	PPARGC1B	peroxisome proliferator-activated receptor gamma, coactivator 1 beta
	KITLG	KIT ligand
	GSK3B	glycogen synthase kinase 3 beta
	APP	amyloid beta (A4) precursor protein
bta-miR-103	BDNF	brain-derived neurotrophic factor
	CLOCK	clock homolog (mouse)
	PTGS2	prostaglandin-endoperoxide synthase 2 (prostaglandin G/H synthase and cyclooxygenase)
bta-miR-106	ABHD5	abhydrolase domain containing 5
	REST	RE1-silencing transcription factor
bta-mi-142–5p	ABCA1	ATP-binding cassette, sub-family A (ABC1), member 1
	ACSL6	acyl-CoA synthetase long-chain family member 6
	CAV2	caveolin 2
	REST	RE1-silencing transcription factor
bta-miR-196a	GLTP	glycolipid transfer protein
bta-miR-296	ABHD4	abhydrolase domain containing 4
	PPARGC1B	peroxisome proliferator-activated receptor gamma, coactivator 1 beta
bta-miR-2368*	ACSL3	acyl-CoA synthetase long-chain family member 3
	CARM1	coactivator-associated arginine methyltransferase 1
	CLOCK	clock homolog (mouse)
	FOXO1	forkhead box O1
	LIF	leukemia inhibitory factor (cholinergic differentiation factor)
	PPARA	peroxisome proliferator-activated receptor alpha
	SNCA	synuclein, alpha (non A4 component of amyloid precursor)
bta-miR-2454	B4GALT1	UDP-Gal:betaGlcNAc beta 1,4- galactosyltransferase, polypeptide 1
	PDGFB	platelet-derived growth factor beta polypeptide

**Table 4:** Functional analysis of gene targets involved in lipid metabolism and adipogenesis.

Category	Function	Pathways	p-value	Predicted gene targets involved
Lipid Metabolism	synthesis	synthesis of lipid	2.15E-15	ABCA1, ABHD5, ACSL3, ACSL6, APP, B4GALT1, BDNF, CAV2, FGF2, FOXO1, GNAI3, KITLG, LIF, PDGFB, PPARA, PTGS2, REST, SNCA, SOCS3
		synthesis of phospholipid	1.68E-06	ABHD5, ACSL6, FGF2, PDGFB, PTGS2, SOCS3
		synthesis of fatty acid	2.07E-06	ABCA1, ACSL3, APP, CAV2, KITLG, LIF, PTGS2, SNCA
		synthesis of steroid	3.27E-06	APP, BDNF, FGF2, KITLG, LIF, PPARA, REST
	metabolism	metabolism of membrane lipid derivative	7.67E-13	ABCA1, ABHD5, ACSL6, APP, B4GALT1, BDNF, FGF2, GNAI3, KITLG, PDGFB, PTGS2, SNCA, SOCS3
		fatty acid metabolism	3.81E-12	ABCA1, ACSL3, ACSL6, APP, B4GALT1, CAV2, FGF2, GLTP, GNAI3, KITLG, LIF, PPARA, PTGS2, SNCA, TEF
		metabolism of phospholipid	3.44E-07	ABHD5, ACSL6, FGF2, PDGFB, PTGS2, SNCA, SOCS3
		metabolism of acylglycerol	5.68E-06	ABHD5, ACSL6, FOXO1, GNAI3, KITLG
	quantity	quantity of lipid	3.73E-11	ABCA1, ADRB1, APP, B4GALT1, BDNF, FOXO1, KITLG, LIF, PPARA, PPARGC1B, PRKCE, PTGS2, SGK1, SLC12A2, SNCA
		quantity of steroid	5.85E-10	ABCA1, ADRB1, APP, BDNF, LIF, PPARA, PPARGC1B, PRKCE, PTGS2, SGK1, SLC12A2
		quantity of triacylglycerol	5.39E-06	BDNF, FOXO1 (includes EG:2308), PPARA, PPARGC1B, PRKCE, PTGS2
		quantity of sterol	7.10E-06	ABCA1, APP, BDNF, PPARA, PPARGC1B, PTGS2
	oxidation	oxidation of lipid	2.84E-08	ABHD5, ACSL3, ACSL6, APP, PPARA, PPARGC1B, PTGS2, SNCA
		oxidation of fatty acid	1.84E-06	ABHD5, ACSL3, ACSL6, PPARA, PPARGC1B, PTGS2
		oxidation of oleic acid	3.36E-06	ACSL3, ACSL6, PPARA
	accumulation	accumulation of lipid	2.47E-07	ABCA1, ABHD5, ACSL6, APP, FOXO1, GSK3B, PAFAH1B2, PPARA
	release	release of lipid	2.97E-07	ABCA1, APP, CAV2, GNAI3, KITLG, PDGFB, PRKCE, PTGS2
		release of fatty acid	1.32E-05	CAV2, GNAI3, KITLG, PDGFB, PRKCE, PTGS2
	cleavage	cleavage of lipid	3.36E-07	ABHD4, ABHD5, GNAI3, PAFAH1B2, PPARA, PTGS2, SNCA
	biosynthesis	biosynthesis of glycosphingolipid	2.25E-06	APP, B4GALT1, FGF2, GNAI3, KITLG
	concentration	concentration of lipid	3.89E-06	ABCA1, APP, CLOCK, PPARA, PTGS2
	hydrolysis	hydrolysis of lipid	4.63E-06	ABHD4, ABHD5, GNAI3, PAFAH1B2, PPARA, SNCA
	esterification	esterification of lipid	5.68E-06	ABCA1, APP, LIF, PPARA
	steroidogenesis	steroidogenesis	1.39E-05	APP, BDNF, FGF2, KITLG, LIF, PPARA
Connective Tissue Development and Function	differentiation	differentiation of adipocytes	1.28E-10	ADRB1, CARM1, FOXO1 (includes EG:2308), GSK3B, LIF, LRP6, PPARA, SMAD7, WNT3A

The qRT-PCR analysis further verified the diet effect on expression of twelve miRNAs, suggesting that that these miRNAs may be involved in regulation of specific functions or pathways in bovine adipose tissue. It is known that various miRNAs regulate adipogenesis [9], [16], [37], [38], [39]. Up to date, the miRNA expression has been widely compared among different species and tissue types. A study on humans showed that miR-103 was the most stable miRNA under different variable conditions, including fat tissue location, body mass index, physiological vs. pathological states, and gender [40]. However, this study did not examine the effect of diet on miRNA expression. In the present study, miR-103 was upregulated in steers fed high fat diet suggesting that a change in dietary fat content may alter the miR-103 expression and play a regulatory role in bovine adipose tissue. This miRNA was found to bind mRNA of caveolin-1, a factor that regulates insulin sensitivity, with the upregulation of miR-103 in liver or fat tissue being associated with impaired glucose homeostasis in mice [41].

In addition, an upregulation of members of the cluster miR-17-92 (miR-19a and -92a) in adipose depots of steers fed high fat diet compared to control was also observed, suggesting an increased adipogenesis in these animals. The miRNA cluster miR-17-92 was shown to be pro-adipogenic as overexpression of miR-17-92 accelerated adipocyte differentiation and increased triglyceride accumulation in 3T3-L1 cells through inhibition of Rb2/p130, a key cell cycle regulator and tumor suppressor [42]. Similarly, expression of other miRNAs (miR-92b, -101, -106, -142-5p, -296) was higher in both fat depots in steers fed high fat diet as compared to control steers, but the regulatory role of these miRNAs remains to be defined in adipose tissue.

To date, the molecular mechanisms of bovine adipogenesis in different adipose depots have not been well defined. Serial analysis of gene expression found a total of 82 genes up or downregulated (>2 fold change) depending on the fat depot (subcutaneous vs. intramuscular fat) in Korean cattle [43]. Differences in gene expression among different fat depots have also been observed in pig and mouse [44]. In this study, we found miRNAs influenced mainly by adipose depot location, such as miR-196a, which was highly expressed in visceral fat while miR-2454 was highly expressed in subcutaneous fat. The adipose depot specificity of these miRNAs is likely related to the regulatory peculiarities of each fat depot. miRNAs tissue specificity has already been reported in other species including mice [45], [46] and human [13] indicating the need of different regulatory mechanisms to address the unique physiology of each tissue type.

Furthermore, it is noticeable to mention that both microarray and qRT-PCR revealed a significant difference of expression of miR-16 in steers fed the two diets. This miRNA has been widely used as an endogenous control miRNA for qRT-PCR analysis [21], [47], [48], [49]. Its expression was not consistent in subcutaneous fat across the two diets. Indeed, miR-16 is reported to regulate a gene involved in apoptosis, B cell lymphoma 2 (BCL2, anti-apoptotic gene) [50], suggesting miR-16 has pro-apoptotic function. Considering that apoptosis is reported to be diet influenced in adipose tissue and associated with obesity [51], we can assume that higher miR-16 levels in steers fed high fat was also diet mediated. This indicates that miR-16 is not a stable endogenous control for adipose tissue across diets that differ in fat level. Therefore, future studies also considering the impact of dietary lipid levels on miRNAs will be needed to identify an alternative endogenous control.

The twelve miRNAs validated by qRT-PCR had at least one predicted gene target with functions related to lipid metabolism and/or adipogenesis. These findings suggest that these differentially expressed miRNAs may play a role in the regulation and development of bovine fat tissue. Some of these miRNAs may even play a broader role such as miR-101 and miR-2368* which are predicted to regulate 7 genes related to lipid metabolism and/or adipogenesis (Table 3), including Peroxisome Proliferator Activated Receptor alpha (PPARA), an important regulator of energy homeostasis in white adipose tissue [52]. The functional analysis indicates that the predicted genes filtered according to their presence in adipose tissue have a broad role in lipid metabolism and adipogenesis. The processes they are involved ranges from synthesis to release of lipids, indicating the important role miRNAs may have on adipose tissue fat metabolism. A previous study using digital gene expression tag profiling showed that genes involved in adipogenesis such as FGF2, GNAI3, LRP6, PAFAH1B2, SMAD7, WNTA3, SOCS3, SGK1, ADRB1, ABHD5, SLC12A2, PRKCE, KITLG, APP, PTGS2, ABCA1, ACSL6, CAV2, ABHD4, LIF, PPARA, SNCA, B4GALT1 and PDGFB were expressed in subcutaneous adipose tissue of beef cattle [5], which belong to the predicted miRNA targets in this study.

It is a complex task to determine the exact role of miRNAs in the gene regulation of adipose as they do not require a perfect complementarity to regulate target messenger RNA [53]. Complementarity is the main feature that miRNA target prediction tools use to find miRNA targets, as a result they may find hundreds of possible targets for one miRNA [54]. Additional studies to experimentally identify the targets of miRNAs differentially expressed in fat tissue will be fundamental to improve our understanding of the roles of miRNAs in gene regulation in adipose tissue under a range of dietary conditions. In addition, the fat tissues collected in this study consist of a mixture of different cell types. Therefore future studies using bovine adipocyte cell lines may further verify the essential regulatory functions of miRNA in bovine adipogenesis.

It is noticeable that miRNAs have also been reported to be part of the regulatory mechanisms of epigenetics. Several DNA methyltransferase genes (DNMTs), which encode proteins responsible for establishment and maintenance of the methylation of the fifth carbon of cytosine residues in DNA, are reported to be targets of miRNAs [55]. Moreover, studies show that DNA methylation and histone modifications not only regulate the expression of protein coding genes, but also of miRNAs [56]. Therefore it is evident the miRNA impact on epigenetic regulatory mechanisms and vice versa. Their interplay modulates gene expression at transcriptional and post-transcriptional levels with implications to the regulation of global gene expression and potentially to adipogenesis. Besides, recently the miR-483-3p was reported to be epigenetically regulated by nutrition during early life in mice and humans with impacts on the regulation of adipose tissue in adult life [57].

In conclusion, the results obtained from this study revealed that the expression of miRNAs differed between subcutaneous and visceral fat depots, suggesting that the molecular mechanism of adipogenesis is site dependent in beef cattle. Our study further identified significant changes in the types and level of expression of miRNAs in steers fed diets differing in fat content. These findings suggest that miRNAs serve as regulators of adipogenesis in response to different dietary conditions. High fat diets have been considered as one of the main factors causing obesity in humans. The identification of the factors that alter miRNAs expression could expand current understanding of the environment and genetic factors that influence gene expression during adipogenesis. Significant individual variation of miRNA expression within the same diet group has been observed. Future studies to link the genotypes to variation of miRNA and their gene targets expressions are essential to elucidate the roles of miRNAs in fat adipogenesis.

## Materials and Methods

### Animal Study and Sample Collections

A total of 16 Hereford×Aberdeen Angus 12 month steers were used in this experiment. They were selected based on similar body weight (∼ 456 kg) and housed in individual pens at the Lethbridge Research Centre and offered feed and water *ad libitum.* The steers were fed experimental diets for approximately 14 weeks. Diets differed in fat content, with the low fat diet containing 1.95% fat, (Control group, n = 8) and the high fat diet containing 5.85% fat. (High fat group, n = 8). Fat content of the diet was increased by including more flaxseed in the diet (Table 5 and Table 6). Experimental diets were fed until slaughter at about 15.5 months of age. Throughout the experiment several performance measures were recorded including body weight gain, feed intake, feed conversion and carcass traits including cutability, backfat thickness and adipocyte size were recorded and reported elsewhere [58], [59]. Subcutaneous fat (backfat) and visceral fat (perirenal fat) were collected from animal carcasses after slaughter, immediately frozen in liquid nitrogen, and kept at −80°C until analyzed. The animal study was approved by the Animal Care Committee of Lethbridge Research Centre, Agriculture Agri-food Canada with ACC# 0930.

**Table 5:** Formulation of Control and High fat diets.

Feed Formulation	Control	High fat
Barley grain, %	85.00	75.00
Barley silage (ave 160), %	10.00	10.00
Vitamin & mineral supplement, %^1^	5.00	5.00
Flax seed, %	0.00	10.00

1 Containing the following minerals and vitamin in 1 kg: 14.67 mg copper, 58.32 mg zinc, 26.73 mg manganese, 0.66 mg iodine, 0.23 mg cobalt, 0.29 mg selenium, 4825 IU vitamin A, 478 IU vitamin D and 32 IU vitamin E.

**Table 6:** Nutritional composition of Control and High fat diets.

Composition	Control	High fat
Dry Matter, %	73.93	74.67
Protein, %	12.71	13.81
Degr. CH2O^1^	47.60	42.00
NEm, Mcal/kg^2^	1.98	2.00
NEg, Mcal/kg^3^	1.33	1.34
Calcium, %	0.62	0.64
Fat, %	1.95	5.85

1 Degradable carbohydrates.

2 Net energy for maintenance.

3 Net energy for gain.

### Measurement of Adipocytes Size

Fat tissues (backfat) were taken by biopsy and were placed in warm saline and transported to the laboratory. Approximately 80 mg of tissue were cut to small pieces and fixed with 1 mL of 5% osmium tetroxide [60] for one week. After removal of the osmium tetroxide solution, fixed tissues were then placed in 8 mol/L urea in physiologic saline to soften the tissue until the most of the adipocytes were isolated. The cells were then washed with saline and were sampled to a 24 well plate for microphotography using an inverted microscope with a digital camera. The diameter of cells was then determined by computer image analysis using software of Motic Images Plus 2.0 ML [61].

### RNA Extraction

Total RNA extraction was performed by homogenizing the fat tissue samples with TRIZOL® (TRI reagent, Invitrogen, Carlsbad, CA, USA) following the manufacturers instructions for samples with high fat content. The concentration of total RNA was measured using the NANODROP® spectrophotometer ND-1000 (Thermo Scientific, Waltham, MA, US) and RNA integrity was measured using the Agilent 2100 BIOANALYZER® (Agilent Technologies Deutschland GmbH, Waldbronn, Germany). RNA with integrity number (RIN) >7.8 was used for miRNA microarray and qRT-PCR analysis.

### Microarray Analysis

MiRNA profiling of adipose tissue samples from visceral and subcutaneous adipose depots from 8 cattle from each diet was performed using AGILENT 8×15 K miRNA array V3 (Agilent Technologies, Santa Clara, CA, USA) customized to profile 672 bovine miRNAs based on the miRBase (Version 15). In brief, total RNA (100 ng) was firstly labeled with the AGILENT miRNA Complete Labeling and Hyb Kit (Version 2.1) by dephosphorylation with calf intestinal phosphatase, followed by denaturing and ligation with Cyanine3-pCp to the 3′ end. The labeled RNA was hybridized with array slides with hybridization buffer and 10× GE blocking agent, and incubated at 55°C for ∼20 hours. Finally, the arrays were washed with GE buffers and scanned at 5 µM resolution on an Agilent G2565CA High Resolution Scanner (Agilent Technologies). Data were processed through Agilent’s Feature Extraction software version 10.7.3.1 using the protocol miRNA_107_Sep09 and the data was normalized to the 75th percentile using GeneSpring GX 11.5 (Agilent Technologies). Differentially expressed miRNAs were filtered by a two sample T-test with standard Bonferroni correction using the Multiple Array Viewer from Multi Experiment Viewer software (v.4.5) [62].

### Dataset

All the microarray data in this study are in compliance to MIAME guidelines and the data have been deposited in the publicly available NCBI’s Gene Expression Omnibus Database (http://www.ncbi.nlm.nih.gov/geo/). The data are accessible through GEO Series accession number GSE35012 (http://www.ncbi.nlm.nih.gov/geo/query/acc.cgi?acc=GSE35012).

### MiRNA Expression Validation by qRT-PCR

Candidate miRNAs were selected based on microarray data for qRT-PCR validation. MiRNA expression was carried out with TAQMAN® miRNA assays according to the manufacturer’s recommendation (Applied Biosystems, Foster City, CA, USA). Briefly, cDNAs were reversely transcribed from 10 ng of total RNA using 5× specific miRNA RT primer and were amplified using a 20× TAQMAN® miRNA assay. Fluorescence signal was detected with an ABI STEPONEPLUS Real-time PCR System detector® (Applied Biosystems). A total of 28 samples from four different treatment combinations (2 diets x 2 tissue types) were used for qRT-PCR analysis, considering 7 biological replicates per group and 3 technical replicates per reaction. Bta-miR-181a was selected as reference miRNA in this study due to its stable expression among all animals and treatments.

### MiRNA Target Prediction and Functional Analysis

The 12 miRNAs selected for qRT-PCR validation were further analyzed to predict which genes they may regulate. Each miRNA was individually searched in the TargetScan Release 6.0. The search was performed for mammals and customized by species (cow/*Bos taurus*). The prediction results were ranked according to context scores and site conservation [63]. The top 100 predictions for each miRNA were analyzed through IPA (Ingenuity® Systems, www.ingenuity.com). The analysis filtered genes expressed only in adipose tissue and this set of genes was submitted to a functional analysis. Right-tailed Fisher’s exact test was used to calculate a p-value determining the probability that each biological function assigned to that data set is due to chance alone.

### Statistical Analysis

The means for performance of steers, fat traits (cutability, backfat thickness and adipocyte size) and average number of miRNAs per individual were compared by a two tailed two sample T-test. The miRNA expressions from qRT-PCR and its respective in microarray were tested for normality and equal variances, submitted to One-way ANOVA and the means were compared by Tukey test. Differences were considered statistically different at p<0.05 and analyses were performed with SAS software (v.9.0).

## Supporting Information

Table S1 The normalized expression of 244 microRNAs detected by microarray in this study is presented for the four experimental groups: Control diet/Subcutaneous fat, High fat diet/Subcutaneous fat, Control diet/Visceral fat and High fat diet/Visceral fat. “UD”, represents the undetected.
(XLSX)
